# Assessment of the Tumbling-Snake Model against Linear and Nonlinear Rheological Data of Bidisperse Polymer Blends

**DOI:** 10.3390/polym11020376

**Published:** 2019-02-20

**Authors:** Pavlos S. Stephanou, Martin Kröger

**Affiliations:** 1 Modeling Department, Novamechanics Ltd., P.O. Box 26014, 1666 Nicosia, Cyprus; 2 Department of Environmental Science and Technology, Cyprus University of Technology, PO Box 50329, 3603 Limassol, Cyprus; 3 Polymer Physics, Department of Materials, ETH Zurich, CH–8093 Zurich, Switzerland

**Keywords:** polymer melt, stochastic differential equation, link tension coefficient, entanglements, bidisperse systems

## Abstract

We have recently solved the tumbling-snake model for concentrated polymer solutions and entangled melts in the academic case of a monodisperse sample. Here, we extend these studies and provide the stationary solutions of the tumbling-snake model both analytically, for small shear rates, and via Brownian dynamics simulations, for a bidisperse sample over a wide range of shear rates and model parameters. We further show that the tumbling-snake model bears the necessary capacity to compare well with available linear and non-linear rheological data for bidisperse systems. This capacity is added to the already documented ability of the model to accurately predict the shear rheology of monodisperse systems.

## 1. Introduction

Understanding the rheological behavior of polymeric systems is of paramount importance in current efforts to improve and optimize upon their processing properties. Polymeric systems of industrial interest are basically never strictly monodisperse, rather they exhibit a distribution of molecular weights. Such a complexity necessitates the modeling of polydisperse polymeric samples.

An important tool that has shown the constitutive maturity and the overwhelming capacity to improve upon our understanding of the rheological behavior of high-molecular-weight (MW) entangled polymer melts and concentrated polymer solutions, both under equilibrium and flow conditions, is the tube-reptation model by de Gennes and Doi & Edwards (DE) [1,2,3]. According to this model, the high-MW chains are confined within an effective mean-field tube constructed by the topological constraints imposed by chains surrounding it. As a result, the test chain may escape the tube only via a one-dimensional diffusion (termed reptation) along the tube’s centerline, which requires, approximately, time equal to the reptation or disengagement time, $\tau_{d}$. Several modifications have emerged over the years in an attempt to deal with remaining shortcomings. Under no-flow conditions, the incorporation of two such mechanisms were (i) contour length fluctuations (CLF), in which one considers the breathing motion of chain ends allowing for a faster, than reptation, relaxation of chain ends, and (ii) constraint release (CR), accounting for the dynamical release of entanglements [2,4,5]. Their incorporation allowed for a quantitative description of the linear viscoelastic (LVE) properties of monodisperse entangled polymer systems [4,5,6,7]. Under flow, the consideration of e.g., chain stretch due to the flow-induced stretching of the tube [8], finite extensibility, [9,10] and convective constraint release (CCR), accounting for an on-average release of entanglements due to flow [10,11,12,13], have improved the comparison with rheological data, although they leave space for further improvement.

The tube-reptation model has also been applied to predicting the rheological behavior of bidisperse polymer blends. Pattamaprom and Larson [14] compared the predictions of the Doi-Edwards-Marrucci-Grizzuti (DEMG) [8,15] and the Mead-Larson-Doi (MLD) [16] (which added the CCR mechanism to the DEMG model) models with their rheological data in start up of steady shearing of concentrated bidisperse polystyrene solutions. They noted that although the MLD model is superior in capturing the steady-state rheological behavior, it predicts lower overshoots in the transient start up of shear than the DEMG model, whose predictions are closer to the observed results. The MLD model has also been noted to be in good agreement with steady state and transient extensional flow experimental data [17]. Leygue et al. [18] developed a model accounting for reptation, CLF, CR, CCR and stretch effects, which they coined the CRAFT (constraint release on average full tensor) model. When a polydisperse system is considered, the CCR effect must be computed from the rate of relaxation of all components. They noted that the model was able to compare well with the shear data of Pattamaprom and Larson [14] and the extension data of Ye et al. [17]. Read et al. [19] extended the GLaMM (Graham, Likhtman & McLeish, Milner) model [20], known to predict the nonlinear shear and extension behavior of entangled solutions of monodisperse linear polymers, to the case of bidisperse polymer melts. The GLaMM model included contributions from reptation, CR, CLF and (in the nonlinear regime) chain stretch. They added that their model compares well against several experimental datasets on bidisperse blends, particularly predicting the onset rate of extension hardening.

A similar, albeit different, formulation aiming to address the rheological response of high MW polymeric melts and concentrated solutions is the model developed by Curtiss & Bird [21] by employing a phase-space formulation within the kinetic theory of undiluted polymers [21,22,23]. We have recently coined it as the tumbling-snake model [24], as it allows for both orientational and curvilinear diffusion of polymer segments. This model entails, as the original tube-reptation model, the solution of a Fokker-Planck (FP) for the single-link distribution function, f(σ,u,t), that describes the probability that at time *t* a chain segment at contour position σ∈[0,1] along the chain is oriented in direction u, with u and σ independent dynamical variables, and |u|=1. Segmental motion is not considered strictly as a one-dimensional reptation along the polymer’s backbone but the chain is also allowed to explore the surrounding space perpendicular to its backbone movements (that we may identify as CR events) controlled by model parameter $\varepsilon^{\prime }\in \left[0,1\right]$. The strictly one-dimensional diffusion process of Doi & Edwards is recovered when $\varepsilon^{\prime }=0$, while $\varepsilon^{\prime }=1$ corresponds to the absence of curvilinear diffusion. Furthermore, the extra (polymeric) stress tensor, see Equation (1) below, contains a term due to the anisotropy of the friction tensor, $\zeta =\zeta_{\mathrm{eq}}\left[\delta -(1-\varepsilon )uu\right]$ involving the, so called, link tension coefficient ε∈[0,1]; if ε=0 there is no friction against motion in the direction u. The stress tensor of the Doi-Edwards model is recovered for ε=0. This is very important as Curtiss and Bird did not employ the concepts of the confining tube and the existence of slip-links in their formulation. Curtiss, Bird and their coworkers solved only the analytically tractable model when $\varepsilon^{\prime }=0$ [21,22,23,25,26].

We have recently solved the tumbling-snake model for $\varepsilon^{\prime }>0$, via the use of Brownian Dynamics simulations, for both steady-state [24,27,28] and time-dependent shear flow [24,28], as well as to steady-state and time-dependent uniaxial [29] and planar elongation [30]. This solution scheme entails the numerical integration of two coupled Itô stochastic differential equations for the variables $U_{t}$ (segment unit vector realization at time *t*) and $\sigma_{t}\in \left[0,1\right]$ (relative contour position at time *t*) (see Equation (5) of Ref. [30]). The only modification to the original tumbling-snake model deemed absolutely necessary was the consideration of a variable link tension coefficient, that vanishes in the absence of flow, and is given by $\varepsilon =\varepsilon_{0}S_{2}^{2}$ [24,28], where $S_{2}$ denotes the 2nd rank uniaxial nematic order parameter of polymer segments [31], and $\varepsilon_{0}$ is a flow-rate independent material constant. This modification has improved upon certain disadvantages of their original model (i.e., exhibiting a constant link tension coefficient): the transient shear and elongational viscosities no longer approach constant values at small times, and spurious time oscillations of the transient second normal stress in startup of shear flow are absent [24,28]. Furthermore, the modified version of the model no longer violates the stress-optic rule, expected to hold close to equilibrium (at larger extensional rates chain stretch becomes non-linear and a failure of the stress-optic rule is expected [32]). It should be stressed that the use of a variable link tension coefficient does not serve to merely amend problematic predictions, but it also bears a microscopic interpretation: When small deformation rates are applied the polymer chain is expected to keep, approximately, its equilibrium configuration and thus the tension stored in each entanglement strand should be the equilibrium one, i.e., ε→0 (see also Example 19.1-1 in Ref. [21]).; the same should hold at early times for any deformation rate [24,28]. This is tantamount to having no friction against motion in the direction u, i.e., as the DE treatment.

These works illustrated that the tumbling-snake model is able of capturing the damping behavior of the transient viscosity in start-up shear experiments at high rates [33,34,35]; at the same time, it correctly predicts the absence of such undershoots in both normal stress coefficients, in line with experimental data [24,28]. This damping behavior has been attributed to the shear-induced rotational motion of chains [28,35], in line with atomistic non-equilibrium molecular dynamics simulations [36,37,38]. As a result, similar undershoots are not expected to occur in elongational flows, due to their irrotational character, which is indeed predicted, without further modifications, by the tumbling-snake model [29,30]. The tumbling-snake model is also able of capturing the peculiar experimental extensional rheological data, according to which the extensional viscosity of polymer solutions is noted to exhibit thinning below the inverse Rouse time and thickening above, whereas the extensional viscosity of polymer melts is monotonically decreasing irrespective of the strain rate [39,40,41], by having the strength $\varepsilon_{0}$ of the link tension coefficient to increase as the polymer concentration decreases [29].

It is worthwhile stressing that the kinetic-theory-based framework employed by Bird et al. [21] is not restricted to entangled polymer melts or concentrated polymer solutions. This is in complete contrast to the tube-reptation theory for which the existence of the mean-field tube necessitates the fact that the MW is beyond the entanglement threshold; strictly speaking, unentangled polymer melts would be confined within a mean-field tube with infinite diameter. On the other hand, the kinetic theory employed by Bird et al. has been used to tackle both dilute polymer solutions and concentrated polymer solutions and polymer melts, see Chapters 17 and 19 of Ref. [21], respectively. Although different mathematical expressions are employed, the same approximations are made for both dilute and undilute polymer chains, with the exception of the mild-curvature approximation (needed to specify that the configuration-space distribution function has significant value only for those configurations in which the chain is approximated by a continuous curve bearing continuous space derivatives, i.e., needed in order to define the single-link distribution function) [21]. This is also exemplified by the comment made by Bird et al. that the parameters employed in their treatment of entangled polymer melts or concentrated polymer solutions, $\varepsilon^{\prime }$ and ε, roughly correspond to the parameters σ and β employed in their kinetic-theory treatment of elastic dumbbells (see Section 13.7 of [21]). Overall, the kinetic-theory treatment employed by Bird et al. offers us the ability to employ a unified approach to study the dynamics of polymeric systems in both their dilute and undilute states.

In industrial applications, monodisperse polymeric samples are never employed. It is for this reason that we attempt in this work to study the rheological response for the second simplest test case, that of a bidisperse melt of linear polymers with two chain lengths, one short and one long, both considered to be long enough to be entangled. Such an endeavor will be vital in our efforts to provide a rigorous modeling of the more intricate case of highly polydisperse industrial polymer resins. Such an undertaking has not been done before in the case of the tumbling-snake model, with the exception of the paper of [42] for the steady-state shear compliance for the analytically tractable case where ($\varepsilon^{\prime }=0$). Schieber [43,44] further considered the use of this model for a polydisperse system (when a Flory-Schulz or a log-normal MW distribution is considered).

The structure of this manuscript is as follows: In Section 2 we revisit the stress tensor of the tumbling-snake model to account for polydispersity [21,42,43,44]. In Section 3, we provide the series expansion of the material functions in the case of steady-state shear for small dimensionless shear rates for comparison with limiting results presented in Section 5. Similarly, in Section 4 we derive analytic expressions for the storage and loss moduli and the complex viscosity. In Section 5 we actually solve the model numerically using data accumulated from the Brownian dynamics simulation executed for the monodisperse samples [24,27,28]. We then conclude with Section 6 where we discuss the significance of this work.

## 2. Stress Tensor

In the case of a polydisperse polymer with “polymerization degree” $N_{a}$ for species *a* related to the number of entanglements, $Z_{a}$, introduced by Doi and Edwards, via $N_{a}=3Z_{a}$, and polymer number density $n_{a}$, the time-dependent (extra or polymeric) stress tensor τ of the tumbling-snake model subjected to a homogeneous flow field characterized by the transposed velocity gradient tensor κ is given by [21,42,43,44]

(1) $$\begin{array}{c} -\frac{\tau (t)}{G}=\sum_{a}w_{a}\left[\left(1-\varepsilon^{\prime }\right)\left({\langle \mathrm{uu}\rangle }_{a}^{\left(1\right)}\left(t\right)-\frac{1}{3}I\right)+3\varepsilon_{0}^{\prime }\left({\langle \mathrm{uu}\rangle }_{a}^{\left(2\right)}\left(t\right)-\frac{1}{18}I\right)+\varepsilon B_{a}\left(t\right)\right], \end{array}$$

with modulus $G=\rho k_{B}T/M_{0}$, temperature *T*, molecular weight corresponding to the portion of the chain corresponding to “one bead” (that, given $N_{a}=3Z_{a}$, can be related to the entanglement molecular weight via $M_{e}=3M_{0}$), gas constant *R*, unit tensor I, mass fraction $w_{a}=n_{a}N_{a}/\left(\sum_{i}n_{i}N_{i}\right)$, coefficients $\varepsilon^{\prime }$ and $\varepsilon_{0}^{\prime }$ interrelated via $\varepsilon_{0}^{\prime }\equiv \varepsilon^{\prime }{\left(N_{a}-1\right)}^{2}$, and a link tension coefficient ε. When the polydisperse system is comprised of components of the same chemical composition the mass and mole fractions are identical to the volume fractions $\phi_{a}$. We consider that $\varepsilon_{0}^{\prime }$ is a universal parameter and does not depend on concentration. As mentioned above, we did not consider a constant ε, as the original treatment of Curtiss & Bird, but related this parameter to the uniaxial order parameter $S_{2}$ [24,28]. We here consider the following generalization to be employed for polydisperse systems:

(2) $$\begin{array}{ccc} \varepsilon & = & \varepsilon_{0}S_{2,\mathrm{av}}^{2},\\ S_{2,\mathrm{av}}^{2} & = & \frac{3}{2}\sum_{a}w_{a}\mathrm{tr}\left({\langle \mathrm{uu}\rangle }_{\mathrm{ani},a}^{\left(1\right)}\cdot {\langle \mathrm{uu}\rangle }_{\mathrm{ani},a}^{\left(1\right)}\right),\\ {\langle \mathrm{uu}\rangle }_{\mathrm{ani},a}^{\left(1\right)} & = & {\langle \mathrm{uu}\rangle }_{a}^{\left(1\right)}-\frac{1}{3}I, \end{array}$$

The stress tensor (1) and the average uniaxial order parameter $S_{2,\mathrm{av}}$ (2) involve the following orientational averages calculated with the solution of the corresponding FP equation [21] for the single-link orientational distribution function $f_{a}\left(\sigma ,u,t\right)$

(3) $$\begin{array}{ccc} {\langle \mathrm{uu}\rangle }_{a}^{\left(1\right)}\left(t\right) & = & \int_{0}^{1}d\sigma \int duf_{a}\left(\sigma ,u,t\right)uu\\ {\langle \mathrm{uu}\rangle }_{a}^{\left(2\right)}\left(t\right) & = & \int_{0}^{1}\sigma \left(1-\sigma \right)d\sigma \int duf_{a}\left(\sigma ,u,t\right)uu,\\ B_{a}\left(t\right) & = & \lambda_{a}\kappa :\int_{0}^{1}\sigma \left(1-\sigma \right)d\sigma \int duf_{a}\left(\sigma ,u,t\right)uuuu, \end{array}$$

where ∫du denotes an integral over the unit sphere, $\lambda_{a}$ a time constant proportional to $\lambda_{0}=\zeta a^{2}/2k_{B}T$, ${\bar{N}}^{1+\beta }$ and $N_{a}^{2}$, where β is a chain constraint exponent, $\bar{N}=\sum_{a}x_{a}N_{a}$ ($x_{a}$ the mole fractions) and κ:uu=(κ·u)·u stands for a two-fold contraction. It should be stressed that now the reptation or disengagement time is not given via $\tau_{d,a}=\lambda_{a}/\pi^{2}$. This is due to the consideration by Doi-Edwards of the form $\tau_{d,a}∼N_{a}^{3}$ whereas the relaxation time selected by Curtiss-Bird also involves $\bar{N}$ in addition to $N_{a}$ [21,42]. Further note that we are adopting throughout the nomenclature of Bird et al. [21], while the τ in (1) is a pressure tensor, and thus the negative stress tensor, in the majority of scientific literature.

## 3. Small Shear Rate Expansion in the Stationary State

Analytical expression of the shear material functions as expansions with respect to the shear rate can be derived analytically following our previous work on monodisperse systems [24,27,28]. Such results are very useful, given that the error bars obtained from Brownian dynamics increase with decreasing rate. The approach to derive analytical results is based on a spherical harmonics expansion of the single-link distribution function around equilibrium. This has already been done previously [24,27,28]; here we employ the expansions available for the orientational averages (3) (see Supplementary Section A of [28] and substitute Wi with $\dot{\gamma }\lambda_{a}$) and use them in the stress tensor expression (1). To readily compare against the available theoretical expressions we will also consider here the constant ε case.

### 3.1. Stationary Regime, Constant ε

Upon inserting the expansions of the orientational averages available in Equation (A1) of the Supplementary Material of Ref. [28] into the stress tensor Equation (1) we obtain the material functions (the shear viscosity, $\eta \equiv -\tau_{yx}/\dot{\gamma }$, and the two normal stress coefficients, $\Psi_{1}\equiv -\left(\tau_{xx}-\tau_{yy}\right)/{\dot{\gamma }}^{2}$ and $\Psi_{2}\equiv -\left(\tau_{yy}-\tau_{zz}\right)/{\dot{\gamma }}^{2}$, respectively) up to second order in $\mathrm{Wi}=\dot{\gamma }\lambda_{L,p}$ where $\lambda_{L,p}=\lambda_{0}N_{L}^{3+\beta }$ is the relaxation time of the pure long component

(4) $$\begin{array}{ccc} \frac{\eta }{G\lambda_{L,p}} & = & \frac{1}{60}\left(1+\frac{2\varepsilon }{3}\right)\frac{\bar{\lambda }}{\lambda_{L,p}}-\frac{2}{245}\left[4\Delta_{3}\left(1-\varepsilon \right)+\frac{23}{3}\Delta_{2}\left(1-\frac{2\varepsilon }{23}\right)\right]\frac{{\bar{\lambda }}_{3}}{\lambda_{L,p}^{3}}{\mathrm{Wi}}^{2},\\ \frac{\Psi_{1}}{G\lambda_{L,p}^{2}} & = & \frac{2\Delta_{1}}{15}\frac{{\bar{\lambda }}_{2}}{\lambda_{L,p}^{2}}-\frac{4}{63}\left(\Delta_{5}+\frac{3}{35}\Delta_{4}-\varepsilon \Delta_{3}\right)\frac{{\bar{\lambda }}_{4}}{\lambda_{L,p}^{4}}{\mathrm{Wi}}^{2},\\ \frac{-\Psi_{2}}{G\lambda_{L,p}^{2}} & = & \frac{4\Delta_{1}}{105}\left(1-\varepsilon \right)\frac{{\bar{\lambda }}_{2}}{\lambda_{L,p}^{2}}-2\left(1-\varepsilon \right)\left(\frac{4\Delta_{4}}{5145}+\frac{\Delta_{5}}{63}-\frac{\Delta_{6}}{3773}\right)\frac{{\bar{\lambda }}_{4}}{\lambda_{L,p}^{4}}{\mathrm{Wi}}^{2}, \end{array}$$

where the coefficients $\Delta_{i}$, i=2,‥,6 are defined in Supplementary Equation (A1b) of Ref. [28] and ${\bar{\lambda }}_{n}=\sum_{a}w_{a}\lambda_{a}^{n}$, with $\bar{\lambda }\equiv {\bar{\lambda }}_{1}$. These expressions match Equations (19.6-13) to (19.6-15) of [21] in the special case $\varepsilon_{0}^{\prime }=0$.

### 3.2. Stationary Regime, Variable ε

If, instead of a constant link tension coefficient, we consider the variable link tension coefficient of the tumbling snake model given by Equation (2), then, up to third order $O({\mathrm{Wi}}^{3})$ we obtain

(5) $$\varepsilon \left(\dot{\gamma }\right)=\varepsilon_{0}\frac{4}{75}\frac{{\bar{\lambda }}_{2}}{\lambda_{L,p}^{2}}{\left(\Gamma_{1}\mathrm{Wi}\right)}^{2},$$

where $\Gamma_{1}$ is a numerical coefficient

(6) $$\Gamma_{1}=12\sum_{\nu =1,\mathrm{odd}}^{\infty }\frac{1}{{\left(\pi \nu \right)}^{2}K_{2}}$$

that first appeared in the Supplementary Equation (A1b) of Ref. [28]. The corresponding steady-state material functions are given, up to $O({\mathrm{Wi}}^{3})$, by

(7) $$\begin{array}{ccc} \frac{\eta }{G\lambda_{L,p}} & = & \frac{1}{60}\frac{\bar{\lambda }}{\lambda_{L,p}}-\frac{2}{245}\left(4\Delta_{3}+\frac{23}{3}\Delta_{2}\right)\frac{{\bar{\lambda }}_{3}}{\lambda_{L,p}^{3}}{\mathrm{Wi}}^{2}+\varepsilon_{0}\frac{2}{3375}\frac{{\bar{\lambda }}_{2}}{\lambda_{L,p}^{2}}{\left(\Gamma_{1}\mathrm{Wi}\right)}^{2},\\ \frac{\Psi_{1}}{G\lambda_{L,p}^{2}} & = & \frac{2\Delta_{1}}{15}\frac{{\bar{\lambda }}_{2}}{\lambda_{L,p}^{2}}-\frac{4}{63}\left(\Delta_{5}+\frac{3}{35}\Delta_{4}\right)\frac{{\bar{\lambda }}_{4}}{\lambda_{L,p}^{4}}{\mathrm{Wi}}^{2},\\ \frac{-\Psi_{2}}{G\lambda_{L,p}^{2}} & = & \frac{4\Delta_{1}}{105}\frac{{\bar{\lambda }}_{2}}{\lambda_{L,p}^{2}}-2\left(\frac{4\Delta_{4}}{5145}+\frac{\Delta_{5}}{63}-\frac{\Delta_{6}}{3773}\right)\frac{{\bar{\lambda }}_{4}}{\lambda_{L,p}^{4}}{\mathrm{Wi}}^{2}, \end{array}$$

or

(8) $$\begin{array}{ccc} \frac{\eta }{\eta_{0,L}} & = & \frac{\eta_{0}}{\eta_{0,L}}-\frac{24}{49}\left(4\Delta_{3}+\frac{23}{3}\Delta_{2}\right)\frac{{\bar{\lambda }}_{3}}{\lambda_{L,p}^{3}}{\mathrm{Wi}}^{2}+\varepsilon_{0}\frac{8}{225}\frac{{\bar{\lambda }}_{2}}{\lambda_{L,p}^{2}}{\left(\Gamma_{1}\mathrm{Wi}\right)}^{2},\\ \frac{\Psi_{1}}{\Psi_{1,0,L}} & = & \frac{\Psi_{1,0}}{\Psi_{1,0,L}}-\frac{10}{21\Delta_{1}}\left(\Delta_{5}+\frac{3}{35}\Delta_{4}\right)\frac{{\bar{\lambda }}_{4}}{\lambda_{L,p}^{4}}{\mathrm{Wi}}^{2},\\ \frac{\Psi_{2}}{\Psi_{2,0,L}} & = & \frac{\Psi_{2,0}}{\Psi_{2,0,L}}-\frac{105}{2\Delta_{1}}\left(\frac{4\Delta_{4}}{5145}+\frac{\Delta_{5}}{63}-\frac{\Delta_{6}}{3773}\right)\frac{{\bar{\lambda }}_{4}}{\lambda_{L,p}^{4}}{\mathrm{Wi}}^{2}, \end{array}$$

As first illustrated by Schieber et al. [21,42], the zero-rate material functions can alternatively be written this way

(9) $$\begin{array}{ccc} \frac{\eta_{0}}{\eta_{0,L}} & = & {\left(\frac{M_{n}}{M_{0}}\right)}^{1+\beta }\frac{M_{w}}{M_{0}}\frac{M_{z}}{M_{0}},\\ \frac{\Psi_{1,0}}{\Psi_{1,0,L}} & = & {\left(\frac{M_{n}}{M_{0}}\right)}^{2+2\beta }\frac{M_{w}}{M_{0}}\frac{M_{z}}{M_{0}}\frac{M_{z+1}}{M_{0}}\frac{M_{z+2}}{M_{0}},\\ \frac{\Psi_{2,0}}{\Psi_{1,0}} & = & -\frac{2}{7}, \end{array}$$

where $M_{n}$ is the number-averaged molecular weight, and $M_{w}$ is the weight-averaged molecular weight. Note that the ratio of the zero-rate viscometric functions is seen not to be affected by polydispersity and is always given as $\Psi_{2,0}/\Psi_{1,0}=-2/7$, independently of $\varepsilon_{0}^{\prime }$ and $\varepsilon_{0}$. We have also used the notation [21,42]

(10) $$M_{z+j}=\frac{\sum_{i}w_{i}M_{i}^{j+2}}{\sum_{k}w_{k}M_{k}^{j+2}},$$

The quantities $M_{z-2}$ and $M_{z-1}$ are equal to $M_{n}$ and $M_{w}$, respectively.

## 4. Linear Viscoelastic Regime

The link tension coefficient vanishes in the linear viscoelastic regime [24,28]. Thus, the expressions for the storage and loss moduli are given as [24]

(11) $$\begin{array}{ccc} G^{\prime }\left(\omega \right) & = & \frac{G}{5}\sum_{a}w_{a}\sum_{\nu =1,\mathrm{odd}}^{\infty }\frac{8K_{2}}{{\left(\pi \nu \right)}^{4}}\frac{{\left(\omega \lambda_{a}\right)}^{2}}{{\left(\omega \lambda_{a}\right)}^{2}+K_{2}^{2}},\\ G^{\prime \prime }\left(\omega \right) & = & \frac{G}{5}\sum_{a}w_{a}\sum_{\nu =1,\mathrm{odd}}^{\infty }\frac{8K_{2}^{2}}{{\left(\pi \nu \right)}^{4}}\frac{\omega \lambda_{a}}{{\left(\omega \lambda_{a}\right)}^{2}+K_{2}^{2}}, \end{array}$$

where $K_{j}=\left(1-\varepsilon^{\prime }\right){\left(\pi \nu \right)}^{2}+j\left(j+1\right)\varepsilon_{0}^{\prime }$. For small frequencies,

(12) $$\begin{array}{ccc} G^{\prime }\left(\omega \right) & = & \frac{G\Delta_{1}}{15}{\left(\omega \bar{\lambda }\right)}^{2},\\ G^{\prime \prime }\left(\omega \right) & = & \frac{G}{60}\omega \bar{\lambda }, \end{array}$$

where $\Delta_{1}\equiv 24\sum_{\nu =1,\mathrm{odd}}^{\infty }\frac{1}{{\left(\pi \nu \right)}^{4}K_{2}}$, whereas for large frequencies,

(13) $$\begin{array}{ccc} G^{\prime }\left(\omega \right) & = & \frac{G}{10}\left[\varepsilon_{0}^{\prime }+2\left(1-\varepsilon^{\prime }\right)\right],\\ G^{\prime \prime }\left(\omega \right) & ∼ & \omega^{-1}, \end{array}$$

Here we have employed Equation (B8) of Ref. [24] and $G^{\prime }\left(\omega \right)/G=\Delta_{1}\left(0\right){\left(\omega \lambda \right)}^{2}/15$, correcting a typo in Equation (B6) of the same reference. The magnitude of the complex viscosity is then given as

(14) $$|\eta^{*}\left(\omega \right)|=\sqrt{{\left[G^{\prime }\left(\omega \right)\right]}^{2}+{\left[G^{\prime \prime }\left(\omega \right)\right]}^{2}}/\omega ,$$

Thus, for small frequencies, $|\eta^{*}\left(\omega \right)|=\eta_{0}$, whereas for large frequencies,

(15) $$|\eta^{*}\left(\omega \right)|=\frac{G}{10\omega }\left[\varepsilon_{0}^{\prime }+2\left(1-\varepsilon^{\prime }\right)\right],$$

Note that at large frequencies both the storage modulus and the magnitude of the complex viscosity become independent of the composition of the polydisperse sample and of all parameters with the exception of $\varepsilon_{0}^{\prime }$ and *G*.

## 5. Brownian Dynamics Results

Having derived analytical expressions for the various regimes, we now turn to the presentation of the full rate-dependent exact numerical results for the tumbling-snake model. Here we merely used the data accumulated via the Brownian dynamics (BD) algorithm that we employed previously for monodisperse samples [24,27]. Further, we focus in the following to bidisperse systems where the two components, termed as the long and the short one, are of the same chemical composition (thus, the volume fraction will be employed instead of the mass fraction).

All figures presented in this manuscript are generated using the variable link tension coefficient; given that the constant ε predictions come with undesirable consequences, we choose not to show these predictions. The analytical results, Equation (8), will be used to test the simulation results, and to extend their validity to “infinitely” small shear rates, where simulation results tend to become more difficult to sample; corresponding figures are depicted in Section 5.2.

### 5.1. Linear Viscoelastic Behavior

**Figure 1:**
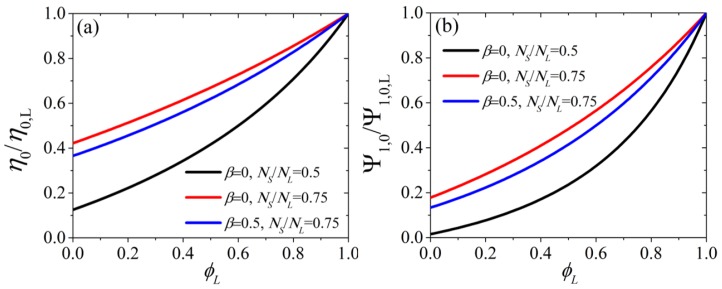
Model predictions for the zero-rate shear viscosity (**a**) and the first normal stress coefficient (**b**), scaled with their corresponding values for the pure long component, for various model parameters as a function of $\phi_{L}$. $N_{S}$ and $N_{L}$ denote the polymerization degree of the short and long component, respectively, and β is the chain constraint exponent.

In Figure 1 we depict how the scaled zero-shear rate viscosity, $\eta_{0}/\eta_{0,L}$, panel (a), and first normal stress coefficient, $\Psi_{1,0}/\Psi_{1,0,L}$, panel (b), vary with the volume fraction of the long chains, $\phi_{L}$, for various values of the parameters β and $N_{S}/N_{L}$. Note that the second normal stress coefficient is equal to $-\left(2/7\right)\Psi_{1,0}$ (Equation (9)) and thus there is no need to depict it separately. We note that under constant β, by increasing the ratio $N_{S}/N_{L}$ both material functions increases as expected, whereas when keeping $N_{S}/N_{L}$ constant and increasing β the material functions decrease. In reality, these two parameters are not to be fitted arbitrarily against experimental data, since $N_{S}$ and $N_{L}$ are dictated by the molecular weight of the two components and chemistry (via $M_{e}$), whereas β can be obtained through the ratio between the zero-rate viscosities of the two components (see Section 5.3). Note that by choosing to scale the first normal stress coefficient with $\Psi_{1,0,L}$ then there is no dependency from the parameter $\varepsilon_{0}^{\prime }$.

**Figure 2:**
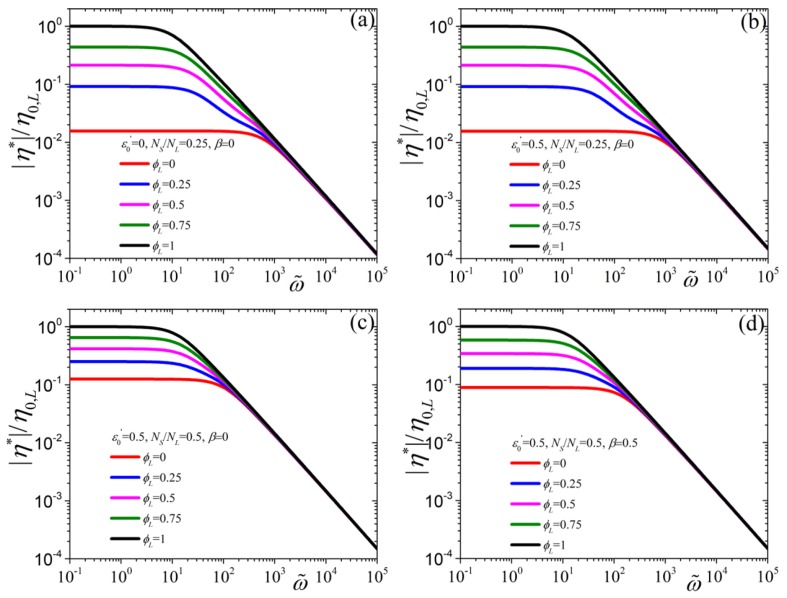
Model predictions for $|\eta^{*}|$, scaled with the zero-rate viscosity of the pure long component, as a function of the dimensionless frequency, $\tilde{\omega }=\omega \lambda_{L,p}$, when (**a**) $\varepsilon_{0}^{\prime }=0$, $N_{S}/N_{L}=0.25$, and β=0, (**b**) $\varepsilon_{0}^{\prime }=0.5$, $N_{S}/N_{L}=0.25$, and β=0, (**c**), $\varepsilon_{0}^{\prime }=0.5N_{S}/N_{L}=\beta =0$, and (**d**) $\varepsilon_{0}^{\prime }=N_{S}/N_{L}=\beta =0.5$, for various values of $\phi_{L}$.

**Figure 3:**
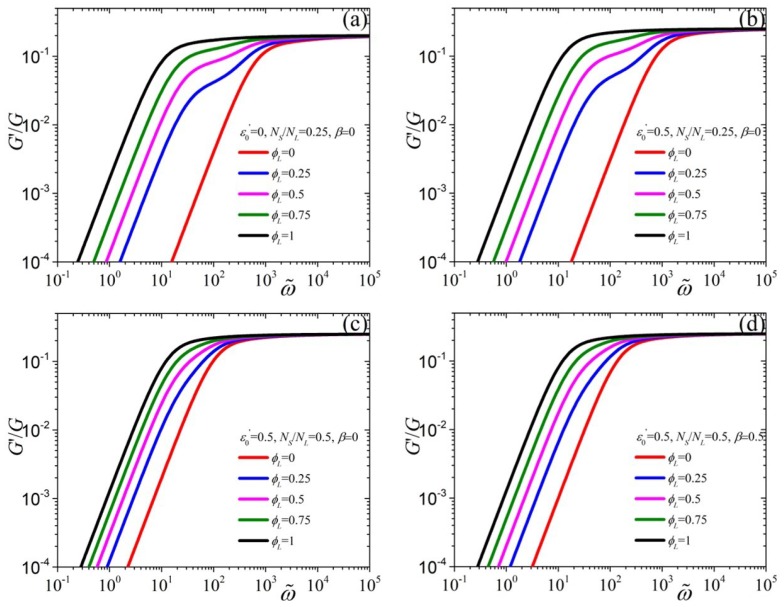
Model predictions for the storage modulus, scaled with *G*, as a function of the dimensionless frequency, $\tilde{\omega }=\omega \lambda_{L,p}$, when (**a**) $\varepsilon_{0}^{\prime }=0$, $N_{S}/N_{L}=0.25$, and β=0, (**b**) $\varepsilon_{0}^{\prime }=0.5$, $N_{S}/N_{L}=0.25$, and β=0, (**c**), $\varepsilon_{0}^{\prime }=0.5N_{S}/N_{L}=\beta =0$, and (**d**) $\varepsilon_{0}^{\prime }=N_{S}/N_{L}=\beta =0.5$, for various values of $\phi_{L}$.

**Figure 4:**
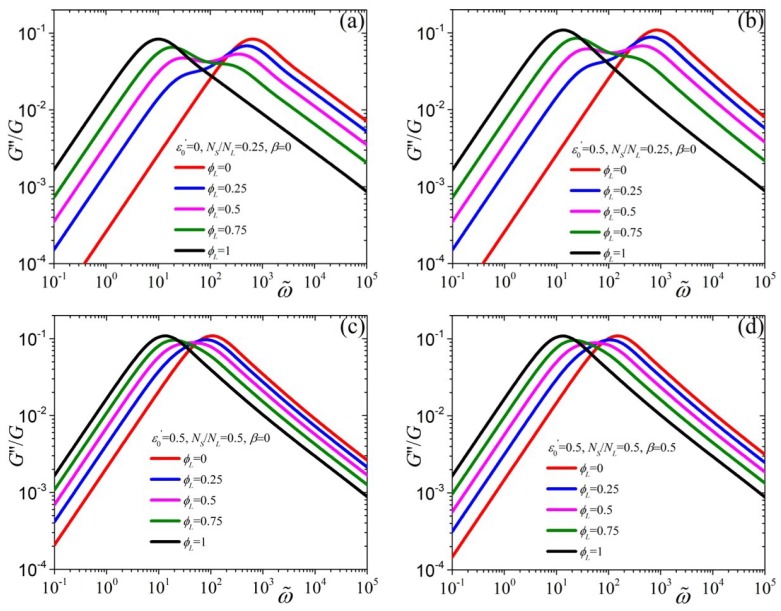
Model predictions for the loss modulus, scaled with *G*, as a function of the dimensionless frequency, $\tilde{\omega }=\omega \lambda_{L,p}$, when (**a**) $\varepsilon_{0}^{\prime }=0$, $N_{S}/N_{L}=0.25$, and β=0, (**b**) $\varepsilon_{0}^{\prime }=0.5$, $N_{S}/N_{L}=0.25$, and β=0, (**c**), $\varepsilon_{0}^{\prime }=0.5N_{S}/N_{L}=\beta =0$, and (**d**) $\varepsilon_{0}^{\prime }=N_{S}/N_{L}=\beta =0.5$, for various values of $\phi_{L}$.

Next, in Figure 2 we investigate the predictions of the tumbling-snake model for $|\eta^{*}|$ as a function of the dimensionless frequency (note that Rouse mode contributions are not included in Figure 2, Figure 3 and Figure 4). Firstly, we should note that at small frequencies the $|\eta^{*}|$ approaches the zero-rate viscosity and thus its value there is dictated by Equation (9). By increasing the value of $\varepsilon_{0}^{\prime }$ from 0 [Figure 2a] to 0.5 [Figure 2b] the $|\eta^{*}|$ curves shift towards larger frequencies, due to the dependency of $K_{2}$ on $\varepsilon_{0}^{\prime }$. On the other hand, by increasing the ratio $N_{S}/N_{L}$ but keeping $\varepsilon_{0}^{\prime }$ constant the zero-rate viscosities, as also alluded to previously, come closer to each other [panel (c)], whereas the opposite occurs when increasing the value of β [panel (d)]. In all cases, however, and irrespective of the values of the parameters, the behavior at large frequencies is seen to be depend only on $\varepsilon_{0}^{\prime }$ and *G* with a power-law exponent equal to −1 and is given by Equation (15).

We turn to the investigation of predictions of the tumbling-snake model for the dimensionless storage modulus, $G^{\prime }/G$, as a function of the dimensionless frequency (Figure 3). As dictated by Equation (12), at small frequencies $G^{\prime }/G$ scales as $∼\omega^{2}$, and by increasing $\phi_{L}$ the curves shift to smaller frequencies. By increasing the value of $\varepsilon_{0}^{\prime }$ from 0 [Figure 3a] to 0.5 [Figure 3b] the $G^{\prime }$ curves shift slightly towards larger frequencies and upwards, due to the dependency of $K_{2}$ on $\varepsilon_{0}^{\prime }$. On the other hand, by increasing the ratio $N_{S}/N_{L}$ but keeping $\varepsilon_{0}^{\prime }$ constant the curves come closer to each other [panel (c)], whereas the opposite occurs when increasing the value of β [panel (d)]. Again, with the exception of $\varepsilon_{0}^{\prime }$ and *G*, as was the case in Figure 2, the behavior at large frequencies is seen to be independent of the parameter values, and is given explicitly by Equation (13).

Finally, in Figure 4 we investigate the predictions of the tumbling-snake model for the dimensionless loss modulus, $G^{\prime \prime }/G$ as a function of the dimensionless frequency. $G^{\prime \prime }$ is proportional to ω at small frequencies. Upon increasing $\phi_{L}$ the curves, as was the case for the storage modulus, shift to smaller frequencies. A second maximum appears at smaller frequencies which becomes more pronounced as $\phi_{L}$ increases. As we increase the value of $\varepsilon_{0}^{\prime }$ from 0 [Figure 4a] to 0.5 [Figure 4b] the $G^{\prime \prime }$ curves shift slightly towards larger frequencies and upwards, whereas, by increasing the ratio $N_{S}/N_{L}$ but keeping $\varepsilon_{0}^{\prime }$ constant, the curves come closer to each other [panel (c)]. Ultimately, by increasing the value of β [panel (d)] the reversed effect takes place.

### 5.2. Non-Linear Regime

**Figure 5:**
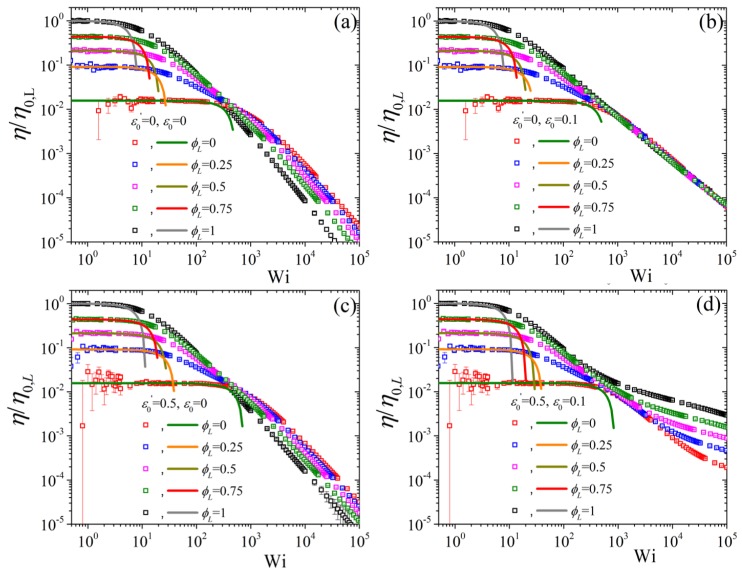
Predictions for the reduced shear viscosity, using the zero-rate viscosity of the pure long component, as a function of dimensionless shear rate and for various values of volume fraction $\phi_{L}$ from our analytical result Equation (8), shown by solid lines up to about Wi = 10, and from the BD simulations (symbols) for (**a**) the DE model ($\varepsilon_{0}^{\prime }=\varepsilon_{0}=0$), (**b**) the analytically solvable Curtiss-Bird model ($\varepsilon_{0}^{\prime }=0,\varepsilon_{0}=0.1$), and the tumbling snake model, when $\varepsilon_{0}^{\prime }=0.5$, and (**c**), $\varepsilon_{0}=0$, and (**d**), $\varepsilon_{0}=0.1$; in all cases $N_{S}/N_{L}=0.5$ and β=0. Note that different colors were employed for the BD simulations (symbols) and for the analytical results at small shear rates (lines) for better visibility.

In Figure 5 we show the predictions of the variation of the shear viscosity, made dimensionless using the zero-shear-rate viscosity of the pure long component, $\eta /\eta_{0,L}$ upon changing the long component volume fraction whilst keeping $N_{S}/N_{L}=0.5$ and β=0. We note that for the original Doi-Edwards model [$\varepsilon_{0}^{\prime }=\varepsilon_{0}=\beta =0$, panel (a)] by increasing the volume fraction the curves move upwards, following Equation (8), and to smaller Wi, since the relaxation time increases as the volume fraction increases. Note that for the pure short component, already large error bars are noted at small shear rates; this trend is to become more severe in the two normal stress coefficients, as shown further below in this section. We should also stress that these theoretical expressions not only provide the zero-shear-rate asymptotes but also the downturn as Wi is further increased. By now increasing $\varepsilon_{0}$ to 0.1 but keeping the non-tumbling case ($\varepsilon_{0}^{\prime }=0$) we note that the predictions in small and intermediate Wi remain unaffected but at large Wi the shear viscosity is now seen to be the same independently of $\phi_{L}$. Furthermore, by keeping $\varepsilon_{0}=0$ and considering the tumbling case ($\varepsilon_{0}^{\prime }=0.5$) we note that the zero-shear limits are kept unchanged, as they should be, but the curves at intermediate and large Wi are seen to shift upwards, leading to larger viscosity values [cf. panels (a) and (b)]. Finally, by having $\varepsilon_{0}^{\prime }=0.5$ and $\varepsilon_{0}=0.1$ once again leaves the small Wi predictions, until about Wi = 100 when $\phi_{L}=1$ and $\mathrm{Wi}=10^{3}$ when $\phi_{L}=0$, unaffected. Since at large shear rates, $\varepsilon \left(\mathrm{Wi}\right)\to \varepsilon_{0}$, the large shear rates power-laws are the same as the one of the tumbling-snake model with a constant link tension coefficient [27].

**Figure 6:**
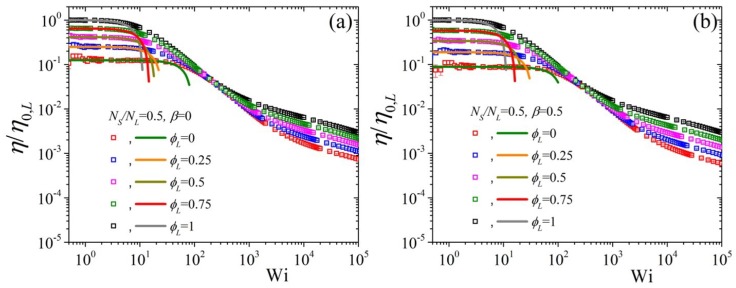
Predictions for the reduced shear viscosity as a function of dimensionless shear rate and for various values of volume fraction $\phi_{L}$ from our analytical result Equation (8) (lines) and from the BD simulations (symbols) for (**a**) β=0 and (**b**) β=0.5, keeping $N_{S}/N_{L}=0.5$, $\varepsilon_{0}^{\prime }=0.5$, and $\varepsilon_{0}=0.1$ constant.

Next, Figure 6 depicts the predictions when keeping $\varepsilon_{0}^{\prime }=0.5$ and $\varepsilon_{0}=0.1$ constant; by changing $N_{S}/N_{L}$ to 0.5 we note that both the low, as dictated by the analytical expression, and high Wi curves come closer to the pure long one [cf. panel (a) with Figure 6d]. On the other hand, by increasing β to 0.5 the exact reverse effect is noted.

**Figure 7:**
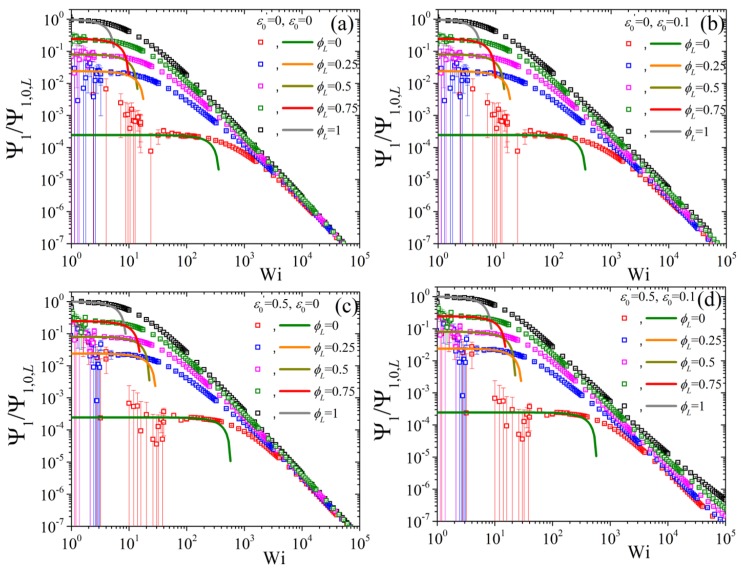
Predictions for the reduced first normal stress coefficient, using the zero-rate first normal stress coefficient of the pure long component, as a function of dimensionless shear rate and for various values of volume fraction $\phi_{L}$ from our analytical result Equation (8) (lines) and from the BD simulations (symbols) for (**a**) $\varepsilon_{0}^{\prime }=\varepsilon_{0}=0$, (**b**) $\varepsilon_{0}^{\prime }=0,\varepsilon_{0}=0.1$, (**c**) $\varepsilon_{0}^{\prime }=0.5$, $\varepsilon_{0}=0$, and (**d**) $\varepsilon_{0}^{\prime }=0.5$, $\varepsilon_{0}=0.1$; in all cases $N_{S}/N_{L}=0.5$ and β=0. Note that different colors were employed for the BD simulations (symbols) and for the analytical results at small shear rates (lines) for better visibility.

In Figure 7 we show the predictions of the variation of the first normal stress coefficient, made dimensionless using the zero-shear-rate first normal stress coefficient of the pure long component, $\Psi_{1}/\Psi_{1,0,L}$ upon changing the long component volume fraction whilst keeping $N_{S}/N_{L}=0.5$ and β=0. We note that in the case of the Doi-Edwards model [panel (a)] the small Wi predictions follow the analytical results of Equation (8) but at large Wi, contrary to the shear viscosity cf. Figure 5a, the predictions are the same irrespective of the volume fraction. The same is noted when having $\varepsilon_{0}=0.1$ [panel (b)], although a slight shift upwards is noted in the intermediate and large shear rates. If we keep $\varepsilon_{0}=0$ and take $\varepsilon_{0}^{\prime }=0.5$ [panel (c)] then the curves move upwards slightly, keeping however their corresponding zero-rate values unaffected, cf. Figure 7a, as was the case for the shear viscosity. However, by having $\varepsilon_{0}^{\prime }=0.5$ and $\varepsilon_{0}=0.1$ [panel (d)] the curves at large Wi are clearly noted to separate.

**Figure 8:**
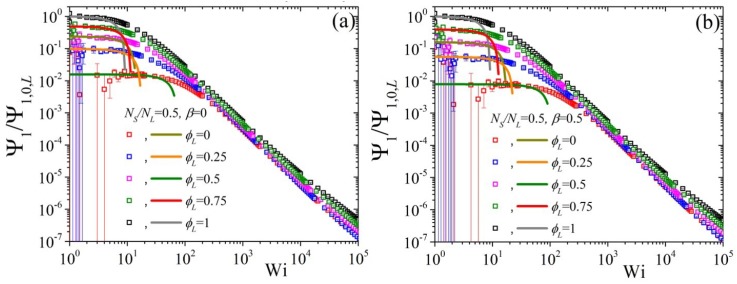
Predictions for the reduced first normal stress coefficient as a function of dimensionless shear rate and for various values of volume fraction $\phi_{L}$ from our analytical result Equation (8) (lines) and from the BD simulations (symbols) for (**a**) β=0 and (**b**) β=0.5, keeping $N_{S}/N_{L}=0.5$, $\varepsilon_{0}^{\prime }=0.5$, and $\varepsilon_{0}=0.1$ constant.

If now we keep $\varepsilon_{0}^{\prime }=0.5$ and $\varepsilon_{0}=0.1$ constant and change $N_{S}/N_{L}$ to 0.5, panel (a) of Figure 8, we note that both the low Wi, as dictated by the analytical expression, and high Wi curves come closer to the pure long one, whereas by increasing β to 0.5 the opposite effect is noted [panel (b)]. These are the same conclusions as the ones reached for the shear viscosity.

**Figure 9:**
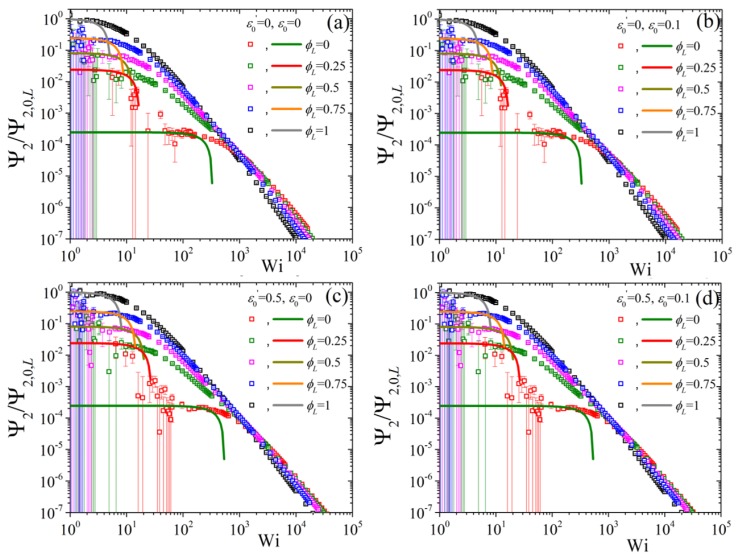
Predictions for the reduced second normal stress coefficient, using the zero-rate second normal stress coefficient of the pure long component, as a function of dimensionless shear rate and for various values of volume fraction $\phi_{L}$ from our analytical result Equation (8) (lines) and from the BD simulations (symbols) for (**a**) $\varepsilon_{0}^{\prime }=\varepsilon_{0}=0$, (**b**) $\varepsilon_{0}^{\prime }=0,\varepsilon_{0}=0.1$, (**c**) $\varepsilon_{0}^{\prime }=0.5$, $\varepsilon_{0}=0$, and (**d**) $\varepsilon_{0}^{\prime }=0.5$, $\varepsilon_{0}=0.1$; in all cases $N_{S}/N_{L}=0.5$ and β=0. Note that different colors were employed for the BD simulations (symbols) and for the analytical results at small shear rates (lines) for better visibility.

**Figure 10:**
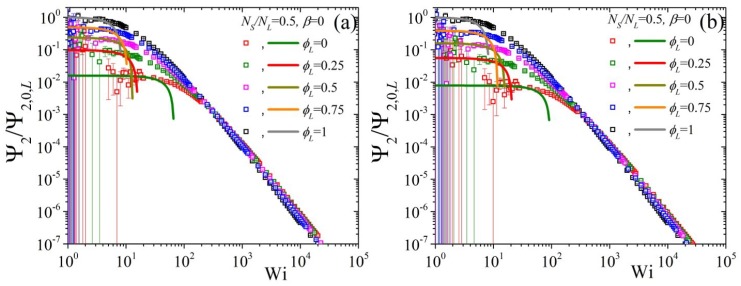
Predictions for the reduced second normal stress coefficient as a function of dimensionless shear rate and for various values of volume fraction $\phi_{L}$ from our analytical result Equation (8) (lines) and from the BD simulations (symbols) for (**a**) β=0 and (**b**) β=0.5, keeping $N_{S}/N_{L}=0.5$, $\varepsilon_{0}^{\prime }=0.5$, and $\varepsilon_{0}=0.1$ constant.

Finally, we conclude by showing the predictions of the variation of the second normal stress coefficient, as $\Psi_{2}/\Psi_{2,0,L}$ as a function of Wi when changing the $\phi_{L}$ but keeping $N_{S}/N_{L}=0.5$ and β=0 (Figure 9). We note that the error bars, especially for the low $\phi_{L}$ curves, are quite substantial, to the point that a clear identification of the zero-rate limits is not possible from the BD simulations alone, making the availability of the analytical results of Equation (8) of paramount importance.At first, we note that by increasing the $\varepsilon_{0}$ from 0 [panel (a)] to 0.1 [panel (b)] very modest changes occur irrespective of the value of $\varepsilon_{0}^{\prime }$ (cf. panel (a) with (b) for $\varepsilon_{0}^{\prime }=0$ and panel (c) with (d) for $\varepsilon_{0}^{\prime }=0.5$). On the other hand, by keeping $\varepsilon_{0}=0$ and changing $\varepsilon_{0}^{\prime }$ from 0 [panel (a)] to 0.5 [panel (c)] leads to a shift of all curves to larger shear rates. Note, however, that the curves at large shear rates are closer to the pure long component one when $\varepsilon_{0}^{\prime }=0.5$. If then we keep $\varepsilon_{0}^{\prime }=0.5$ and $\varepsilon_{0}=0.1$ constant and change $N_{S}/N_{L}$ to 0.5, panel (a) of Figure 10, we note that that the low, as dictated by the analytical expression, Wi curves come closer to the pure long one, whereas by increasing β to 0.5 the opposite effect is noted [panel (b)]. On the contrary, the predictions at large shear rates are seen to be almost unaffected by these changes.

### 5.3. Comparison with Experimental Data

**Figure 11:**
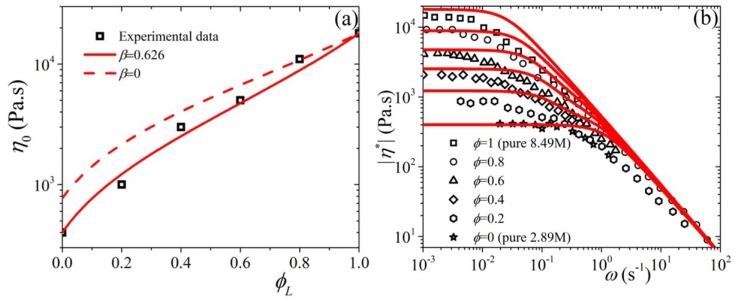
Comparison of the predictions of the tumbling-snake model (lines) against experimental data (symbols) [14] for (**a**) the zero-shear viscosity (where also the prediction for β=0 is shown), and (**b**) the $|\eta^{*}|$; the parameter values are $N_{L}=42$, $N_{S}=14.7$, β=0.626, $\eta_{0,L}=1.8\times 10^{4}$ Pa·s, $\varepsilon_{0}^{\prime }=10^{-3}$ and $\lambda_{L,p}=450$ s (the latter two needed only for $|\eta^{*}|$).

**Figure 12:**
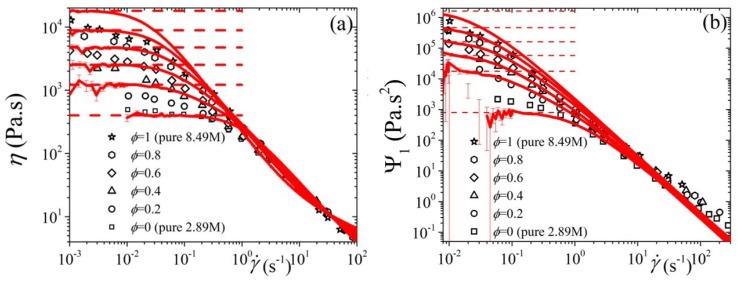
Comparison of the predictions of the tumbling-snake model (lines) against experimental data (symbols) [14] for (**a**) the shear viscosity, and (**b**) the first normal stress difference, as a function of shear rate; in addition to the parameter values mentioned in Figure 11, a volume-fraction dependent $\varepsilon_{0}$ is employed of the form $\varepsilon_{0}=0.18\left(1-2\phi_{L}/3\right)$.

In this section, we compare model predictions against the linear and non-linear rheological data of Pattamaprom and Larson [14] for highly entangled bidisperse polystyrene (PS) solutions. These solution were at 7% volume fraction in the solvent tricresyl phosphate (TCP). The molecular weight is $M_{L}=8.42\times 10^{6}$ ($M_{w}/M_{n}=$ 1.14) for the long component, and $M_{S}=2.89\times 10^{6}$ ($M_{w}/M_{n}=$1.09) for the short component. All rheological measurements presented below are depicted at 40 ${}^{\circ }$C. Following Pattamaprom and Larson, the value for the entanglement molecular weight in the solution was estimated from the entanglement molecular weight in the melt via [45] $M_{e}^{\mathrm{solution}}=M_{e}^{\mathrm{melt}}\theta^{-4/3}$, where θ is the polymer volume fraction in the solution; for the PS solution data of Pattamaprom and Larson θ=0.07. Thus, using a value of $M_{e}^{\mathrm{melt}}=17,000$ gr/mol [45], we obtain $M_{e}^{\mathrm{solution}}=5.89\times 10^{5}$ gr/mol or that $Z_{L}=14$ and $Z_{S}=4.9$ and $N_{L}=42$ and $N_{S}=14.7$. Given that for the pure components, $\eta_{0,L}/\eta_{0,S}={\left(N_{L}/N_{S}\right)}^{3+\beta }$ and the values of the zero-rate viscosities of the two components, $\eta_{0,L}=1.8\times 10^{4}$ Pa·s and $\eta_{0,S}=400$ Pa·s [14], we easily obtain the value of β=0.626 and directly employ the value of $\eta_{0,L}=1.8\times 10^{4}$ Pa·s, which is seen to accurately predict the behavior also for $0<\phi_{L}<1$ [see Figure 11a]. The value for $\varepsilon_{0}^{\prime }=10^{-3}$ is obtained by noting the power-law of the shear viscosity at large shear rates (equal to −0.75) and referring to Figure 4 of Ref. [27] (for $\varepsilon_{0}>0$). The relaxation time of the pure long component, $\lambda_{L,p}=450$ s is obtained by properly shifting the predictions of the model for $|\eta^{*}|$ [Figure 11b] and the non-linear material functions (Figure 12). Finally, to accurately compare with the predictions of the shear viscosity we need to employ a volume-fraction dependent $\varepsilon_{0}$ of the form, $\varepsilon_{0}=0.18\left(1-2\phi_{L}/3\right)$. We had reached the same conclusion recently when we needed to have the value of $\varepsilon_{0}$ to increase as the polymer concentration, in polymer solutions, decreases to match the behavior of the stationary extensional viscosity of PS polymer solutions [29]. This is tantamount to having the friction tensor become more isotropic with decreasing polymer concentration. The same conclusion could be extended to bidisperse melts if we identify the short component as acting as a solvent for the long one. This is actually the same as our previous treatment mentioned above to match the behavior of the stationary extensional viscosity of PS polymer solutions since the solvent employed by Huang et al. [40] in their rheological measurements is a styrene oligomer with a MW far below the entanglement MW.

Overall, the predictions of the tumbling-snake model, both in the linear and the non-linear regime, are noted to compare well with the experimental data. And it does so as well as other tube models employed in the literature (see Section 1). We should here mention that the original Curtiss-Bird model, i.e., the non-tumbling version with $\varepsilon_{0}^{\prime }=0$, fails to capture the rheological behavior at large shear rates since for ε>0 it predicts a constant power-law exponents of −1 and −2 for the viscosity and first normal stress coefficient, respectively, whereas the experimental data exhibit power-law exponents of −0.75 and −1.75, respectively. Further, the use of a constant ε value would lead to unfavorable prediction in the transient behavior, despite having not showing it here.

## 6. Conclusions

In this work, we discussed the features of the tumbling-snake model for entangled bidisperse polymer melts subjected to steady-state shear flow. Following our recent work [24,28], we employ a variable link tension coefficient, given by $\varepsilon =\varepsilon_{0}S_{2,\mathrm{av}}^{2}$ [see Equation (2)], which has been noted to amend several shortcomings of a constant link tension coefficient originally suggested by Bird et al. [21,25,26]. To the best of our knowledge, even the non-tumbling version of the model ($\varepsilon_{0}^{\prime }=0$) has not been presented before in the case of a bidisperse system; Schieber [43,44] had only provided the predictions for a polydisperse system. We have shown that the low shear rate predictions are in accord with the theoretical results, Equation (8), which is important, particularly for the normal stress coefficients, to identify accurately the zero-rate values, which are noted to depend strongly on both the volume fraction of the long component, $\phi_{L}$, and the ratio between the number of entanglements of the two components, $N_{S}/N_{L}$. On the other hand, the predictions at large shear rates are seen, in many cases, to be independent of these parameters. We have further shown that the predictions of the tumbling snake model are in very good agreement with rheological data for highly entangled bidisperse PS solutions in both the linear and non-linear regimes [14]. The model is as successful in predicting the linear and non-linear rheological characteristics of bidisperse polymer blends as many tube models employed in the past (see Section 1). These models all include the notions of reptation, CLF, CR, CCR and stretch effects. Our current version of the tumbling snake model only includes reptation (the first term on the right-hand side of Equation (1) in Ref. [28]) and CR effects (the second term on the right-hand side of Equation (1) in Ref. [28]) via the orientational diffusion term in the single-link distribution function evolution equation.

In conclusion, the tumbling-snake model, which amends the problems of the original Curtiss-Bird model by employing a variable link-tension coefficient, has been shown able of providing a very adequate description of the available rheological measurements of monodisperse entangled polymer melts and concentrated polymer solutions when subjected to shear [24,27,28], uniaxial elongation [29], and planar elongation [30], and, in this work, for entangled bidisperse polymer blends when subjected to shear. Solving it necessitates the use of very simple Brownian dynamics simulations coupled with the analytical predictions at small rates. By further introducing additional refinements, such as CLFs (see e.g., [6,46,47] and references therein), flow-induced alignment of chain ends [31,48], chain stretch [8,49], and convective constraint release [10,11,12], could further improve the tumbling-snake’s model capacity to quantitatively predict the rheological response of entangled polymer melts and concentrated polymer solutions. Especially for the latter two, special attention should be sought to derive the evolution equation for the single-link distribution function properly via the use of non-equilibrium thermodynamics, particularly building upon the work of Öttinger [50].
